# The Anticancer Effect of Kaempferol Through Downregulation of CDKs and PD-L1 in Triple-Negative Breast Cancer Cells

**DOI:** 10.3390/cancers17243911

**Published:** 2025-12-06

**Authors:** Sukhmandeep Kaur, Patricia Mendonca, Shubham D. Mishra, Karam F. A. Soliman

**Affiliations:** 1 Division of Pharmaceutical Sciences, Institute of Public Health, College of Pharmacy and Pharmaceutical Sciences, Florida A&M University, Tallahassee, FL 32307, USA; sukhmandeep1.kaur@famu.edu (S.K.); shubham1.mishra@famu.edu (S.D.M.); 2 Department of Biology, College of Science and Technology, Florida A&M University, Tallahassee, FL 32307, USA

**Keywords:** kaempferol, triple-negative breast cancer, PD-L1, CCL2, TGM2, CDKs

## Abstract

**Background:** Triple-negative breast cancer (TNBC) is an aggressive subtype of breast cancer characterized by poor prognosis, high metastatic potential, and limited targeted therapies. **Methods:** This study evaluates the anticancer activity of kaempferol in two genetically and ethnically distinct TNBC models, MDA-MB-231 (Caucasian) and MDA-MB-468 (African American) cells, utilizing cell viability, cell proliferation, apoptosis, cell cycle arrest, qPCR, and Abby analysis assays. **Results:** Kaempferol inhibited TNBC cell viability and proliferation in a dose- and time-dependent manner, in both 2D monolayer and three-dimensional (3D) cell cultures, and induced apoptosis and S phase cycle arrest. This investigation demonstrated that kaempferol affects cancer cells by downregulating CDK1, CDK4, CDK6, and CDK7 at both the gene and protein levels in MDA-MB-231 cells, and specifically CDK6 and CDK7 in MDA-MB-468 cells. It reduced the expression of PD-L1, JAK1, STAT3, MUC-1, and NF-κB1 (p50), indicating that the underlying pathway of PD-L1 inhibition may involve targeting the JAK1/STAT3 pathway and downregulating MUC-1, while inhibiting NF-κB1 levels. Kaempferol also inhibited the overexpression of CCL2 and TGM2, which are responsible for the development of resistance to targeted therapies, such as anti-PD-L1 immunotherapy, in TNBC. **Conclusions:** Kaempferol acts as a multi-target modulator of CDKs and PD-L1-associated signaling in TNBC, extending prior reports of its antiproliferative and pro-apoptotic effects in MDA-MB-231 and, significantly, providing comparative evidence across MDA-MB-231 versus MDA-MB-468 models. Since the levels of PD-L1 have been previously shown to be regulated by CDKs, this study is the first to report the dual inhibitory effect of a flavonoid of natural origin on CDKs and PD-L1 levels in TNBC cells.

## Simple Summary

Triple-negative breast cancer is an aggressive form of breast cancer that mainly affects younger women and has limited targeted treatment options. Patients from different racial and genetic backgrounds can also respond differently to treatment, which contributes to outcome disparities. This study explored the natural compound kaempferol, commonly found in fruits and vegetables, as a potential therapeutic option, using two genetically distinct triple-negative breast cancer lines derived from Caucasian and African American women. The results showed that kaempferol can reduce cancer cell growth, trigger cancer cell death, and interfere with the proteins that control cancer cell division and immune resistance. These findings suggest that kaempferol may enhance treatment outcomes by simultaneously targeting multiple cancer-related pathways. This study provides new evidence supporting the use of natural compounds as promising options to improve therapy response in aggressive breast cancers.

## 1. Introduction

Triple-negative breast cancer (TNBC) is one of the most aggressive and non-responsive subtypes regarding standard treatment options for breast cancer. TNBC tumor cells lack estrogen receptors (ERs), progesterone receptors (PRs), and human epidermal growth factor receptor 2 (HER-2), making them unresponsive to standard hormonal and targeted therapies. TNBC accounts for about 10–15% of all human breast cancers, and it is more aggressive than other subtypes [1]. It is more likely to occur in younger women, and most patients report recurrence and metastasis within 3 years of the first diagnosis. Among ethnic groups, African American and Hispanic women are considered high-risk groups. While the incidence is highest in Caucasians, the disease shows the worst prognosis in African American women [1].

Due to the heterogeneity of TNBC, therapeutic strategies that are more specific in targeting the complex interplay between cancer cells and the tumor immune microenvironment (TIME) are needed [2]. Recent advances in TNBC treatment have shifted toward immune checkpoint blockade and toward reversing reduced T cell infiltration at the tumor site by targeting the interaction between programmed cell death protein 1 (PD-1) and programmed cell death protein ligand 1 (PD-L1) [3]. The successful clinical performance of these antibody-based immune therapies has further highlighted the potential to target unique subtypes of the TIME, thereby increasing the responsiveness of immunotherapeutic treatments. This highlights the dire importance of a deeper understanding of time and the development of therapeutic approaches to overcome resistance to targeted therapies, such as immune checkpoint inhibitors [2].

Within the TIME, CCL2/MCP-1 (Chemokine (C-C Motif) Ligand 2/Monocyte Chemotactic Protein-1) is a molecule of interest for therapeutic intervention, belonging to the family of chemokines that regulate the recruitment of macrophages to the inflammatory site [4]. The CCL2-induced recruitment of macrophages to the tumor microenvironment reduces the efficacy of PD-L1/PD-1 inhibitors by delaying T-cell infiltration into the tumor. Moreover, the transcription factor NF-κB, a key regulator of immunity and inflammation, has emerged as a positive regulator of PD-L1 by binding to its promoter post-transcriptionally and modulating various pathways [5,6,7]. Numerous studies have linked NF-κB activation to the development of resistance and a decline in immune function. With established evidence of PD-L1’s role in reduced immune function and increased resistance, it is crucial to explore the effect of targeting NF-κB-mediated PD-L1 expression in resistant TNBC tumors [6]. Additionally, Transglutaminase-2 (TGM2), which is widely distributed in various tissues, is a transpeptidase enzyme that participates in signal transduction by activating GTPase enzymes and in protein cross-linking. It exerts its functions in both normal and inflamed as well as cancer cells [8]. TGM2 is minimally expressed in normal epithelial tissues but is upregulated in tumor cells, where it is involved in cell survival, tumor invasion, and motility. Interestingly, TGM2 can activate the PI3K/AKT pathway, inducing the levels of both PD-L1 and CCL2. Choi et al. (2020) reported that, in addition to inhibiting CCL2, TGM2 inhibition can also reverse the development of resistance to PD-L1 immunotherapy [8].

Given the complexity of TIME and its essential role in cancer development and progression, natural compounds have been extensively studied for their potential anticancer activity, targeting multiple molecular targets to exert anti-inflammatory, antioxidant, and antiproliferative effects [3]. Additionally, based on extensive literature, natural compounds are safer, more affordable, and readily available as treatment options for several types of human cancer. Kaempferol is a flavonoid of natural origin, commonly found in tea, tomato, green peas, broccoli, and berries, and has been reported to have extensive anticancer activity. It exerts its anticancer effects by targeting multiple molecular pathways, including the cell cycle, cell proliferation, apoptosis, autophagy, and the inhibition of metastasis and angiogenesis [7,9,10,11], demonstrating significant potential for cancer prevention and therapy.

This study aimed to explore the anticancer mechanisms of kaempferol by modulating the TIME components and demonstrate its potential for use in combination with immunotherapy to reduce treatment resistance. The experiments were conducted using genetically distinct TNBC cell lines: MDA-MB-231 and MDA-MB-468.

## 2. Materials and Methods

### 2.1. Reagents

Kaempferol (≥98% purity, HPLC grade), Alamar Blue^®^, dimethyl sulfoxide (DMSO), chloroform, interferon-gamma (IFN-γ), and isopropyl alcohol were obtained from Sigma-Aldrich (St. Louis, MO, USA). Heat-inactivated fetal bovine serum (FBS-HI), high-glucose Dulbecco’s Modified Eagle Medium (DMEM), penicillin–streptomycin, and phosphate-buffered saline (PBS) were sourced from Genesee Scientific (San Diego, CA, USA). TRIzol reagent for RNA isolation was purchased from ThermoFisher Scientific (Wilmington, DE, USA). SYBR Green master mix, the iScript Advanced cDNA synthesis kit, and gene-specific primers were obtained from Bio-Rad (Hercules, CA, USA). Protein concentrations were determined using the Bicinchoninic Acid (BCA) Assay Kit (Item #23225). All Simple Western reagents and assay plates for the Abby™ system were supplied by ProteinSimple (San Jose, CA, USA), and primary antibodies were acquired from Cell Signaling Technology (Danvers, MA, USA). ELISA kits for PD-L1 (Cat# ELH-B7H1-1), CCL2 (Cat# ELH-MCP1), and TNF-α were purchased from RayBiotech (Norcross, GA, USA).

### 2.2. Cell Culture

TNBC cell lines MDA-MB-231 and MDA-MB-468 obtained from the American Type Culture Collection (ATCC, Manassas, VA, USA) were maintained in DMEM supplemented with 10% FBS-HI and 1% penicillin–streptomycin and cultured at 37 °C in a humidified incubator with 5% CO_2_. Cells were routinely expanded in T-75 flasks and sub-cultured upon reaching approximately 90% confluence. For all experimental procedures, the culture medium was replaced with DMEM containing 2.5% FBS-HI and no antibiotics.

### 2.3. Cell Viability

Cell viability was assessed in both 2D and 3D cell cultures using the Alamar Blue assay with MDA-MB-231 and MDA-MB-468 cells at varying kaempferol concentrations: 1.56–200 µM for 2D and 25–200 µM for 3D. In the 2D cell viability assay, cells were seeded at a density of 3 × 10^4^ cells/100 µL/well in a tissue culture-treated 96-well plate and treated with kaempferol the following day. They were then incubated for 24 and 48 h. In the 3D cell viability assay, Thermo Fisher Scientific Nunclon Sphera 96-well bottom plates were used. The cells were plated at a seeding density of 3 × 10^4^ cells in 100 μL/well and incubated for 48 h to form spheroids. Once the spheroids were formed in 3D, the cells were treated with kaempferol concentrations (25 to 200 μM) and incubated for 24 h. Then, the Alamar Blue solution was added to the cells and incubated for 4 h. The fluorescence changes in the wells (in both 2D and 3D) were measured at an excitation/emission wavelength of 530/590 nm using an Infinite M200 microplate reader from Tecan Trading AG (Morrisville, NC, USA). The fluorescent signal was proportional to the number of viable cells in each treatment.

### 2.4. Cell Proliferation Assay

Cell proliferation was assessed using the Alamar Blue assay. TNBC cell lines (MDA-MB-231 and MDA-MB-468) were seeded at a density of 5 × 10^3^ cells per well in tissue culture-treated 96-well plates and incubated overnight. After 24 h, the cells were treated with varying concentrations of kaempferol (3.12 to 200 µM), Taxol (a standard chemotherapeutic agent), or DMSO (vehicle control) for 48, 72, and 96 h. After the treatment period, Alamar Blue reagent was added to all wells, and the mixture was incubated for 4 h at 37 °C in a 5% CO_2_ atmosphere. The resulting fluorescence signal was then recorded using an Infinite M200 microplate reader (Tecan Trading AG, Morrisville, NC, USA) at excitation and emission wavelengths of 530 nm and 590 nm, respectively.

### 2.5. Apoptosis Assay

Apoptosis was assessed using the Annexin V/PI staining assay in both MDA-MB-231 and MDA-MB-468 cells after exposure to kaempferol. Cells were plated at a density of 1 × 10^6^ cells per well in 6-well plates and allowed to adhere overnight. The next day, the cultures were treated with increasing concentrations of kaempferol (12.5–100 µM), while control wells received <0.1% DMSO. After a 24 h incubation period, both control and treated cells were harvested by trypsinization, centrifuged, and washed once with PBS. Cell pellets were then resuspended in 500 µL of the assay binding buffer supplied with the kit and stained with 5 µL Annexin V and 10 µL PI for 10–15 min, following the manufacturer’s instructions. Samples were subsequently acquired on a Sony SH800 cell sorter (San Jose, CA, USA) at a final event count of 1 × 10^4^ cells per sample, and data were processed using CELLQuest Pro (Version 5.1).

### 2.6. Dual Acridine Orange/Ethidium Bromide (AO/EtBr) Fluorescent Staining Assay

The AO/EtBr assay was used to confirm apoptosis induction in kaempferol-treated MDA-MB-231 and MDA-MB-468 cells in both 2D and 3D cultures. For 2D cells, a density of 3 × 10^4^ cells/well was seeded in a tissue culture-treated 96-well plate and incubated at 37 °C in 5% CO_2_ for 24 h. In 3D culture, cells were seeded in ThermoFisher Scientific Nunclon Sphera 96U bottom plates at a seeding density of 3 × 10^4^ cells per well and incubated at 37 °C in 5% CO_2_ for 48 h. After incubation, the cells were treated with different concentrations ranging from 25 µM to 100 µM and incubated for 24 h. Then, 5 µL of dual fluorescent staining solution containing 100 µg/mL AO and 100 µg/mL EB (AO/EB, Sigma, St. Louis, MO, USA) was added to all wells, and the wells were washed five times with PBS. The fluorescent staining was then analyzed using an Olympus Cell Sens Standard Cytation 5 cell Imaging reader (BioTek Instruments, Inc., Winooski, VT, USA).

### 2.7. Cell-Cycle Assay

The effect of kaempferol on DNA content and cell cycle distribution was assessed using a cell cycle arrest assay. The TNBC cells were seeded in T-25 flasks and treated with different concentrations of kaempferol (3.125 µM to 50 µM) the following day. The cells were harvested after 24 h of incubation, and the cell pellets were collected. The cells were resuspended and fixed using ice-cold PBS and 70% ethanol. Following this, the cell pellets were gently resuspended in 300 µL of propidium iodide (PI) + RNase staining solution, followed by incubation at 37 °C in the dark for 30 min. The cell samples were then analyzed using a Sony SH800 Cell Sorter (San Jose, CA, USA) flow cytometer to investigate the distribution of cells across different cell cycle phases.

### 2.8. qPCR

The iScript advanced reverse transcriptase enzyme from Bio-Rad was used for cDNA synthesis, and RT-PCR was performed using target genes underlying cell cycle arrest. Briefly, the cells were seeded in T-75 flasks followed by incubation for 24 h, and treated with DMSO, kaempferol at concentrations of 25 µM and 50 μM for MDA-MB-231 and MDA-MB-468 cells, respectively, TNF-α (50 ng/mL) or IFN-γ (100 ng/mL), and co-treated with kaempferol + TNF-α (50 ng/mL) or IFN-γ (100 ng/mL), according to each experiment. In the co-treatment with kaempferol and TNF-α or IFN-γ, the cells were pre-treated for 1 h before kaempferol treatment. After incubation for 24 h, the cells were harvested and pelleted.

TNF-α (50 ng/mL) was used in selected experiments to induce pathological upregulation of CDKs, CCL2, and TGM2. TNF-α is a well-established inflammatory cytokine that activates multiple pro-proliferative pathways (including NF-κB and MAPK), leading to the transcriptional elevation of CDK1, CDK4, CDK6, CDK7, CCL2, and TGM2 in TNBC models [12,13]. Similarly, IFN-γ (100 ng/mL) was used to induce PD-L1 expression and its associated markers, as this cytokine is physiologically secreted by activated CD8^+^ T cells and is the primary driver of PD-L1 transcription in tumor cells via the JAK/STAT axis [14].

The cell pellets were then processed for RNA isolation using the TRIzol method. The isolated RNA was quantified using a Nanodrop, and cDNA was synthesized using iScript Advanced Transcriptase based on the RNA concentration. In this, the 5X iScript advanced reaction mix, reverse transcriptase, sample RNA, and water were combined in a 0.2 mL tube, and the reverse transcription reaction was performed using the thermocycler at 46 °C for 20 min and 95 °C for 1 min in 40 cycles. For RT-PCR amplification, an SYBR Green kit was used, and the cDNA was amplified according to the protocol with primers specific to the gene of interest. The mixture of cDNA, SYBR green, master mix, and water was processed on the Bio-Rad CFX96 Real-Time System (Hercules, CA, USA). The thermal cycling protocol included an initial holding step at 95 °C for 2 min and denaturation at 95 °C for 15 s, followed by 40 cycles of 60 °C for 30 s (annealing/extension) and 60 °C for 5 s/step (melting curve). Information on the primers used is provided in Supplementary Table S1.

### 2.9. Abby Protein Analysis

To determine protein expression, the cells were treated with TNF-α, kaempferol, or both TNF-α and kaempferol. For the co-treatment, TNF-stimulated cells were treated with different concentrations of kaempferol 1 h after TNF-α addition. After 24 h of incubation, the cells were harvested, washed, and pelleted. A protease inhibitor-containing lysis buffer was added to these pellets, and the cells were then sonicated and centrifuged. The supernatant was subsequently collected. The samples were then analyzed for protein concentration using the Bicinchoninic Acid (BCA) Protein Assay Kit according to the manufacturer’s protocol. Protein expression was obtained with the automated AbbyTM ProteinSimple (San Jose, CA, USA). The analysis was performed using plates and reagents provided by ProteinSimple, and the protein samples and antibodies were optimized by testing different concentrations and dilutions. Protein samples were diluted with 0.1× sample buffer to achieve a final concentration of 1 to 2 mg/mL total protein. The samples were heated at 95 °C for 5 min. Then, the primary antibody (dilution 1:125), secondary antibody, chemiluminescent substrate, blocking solution, and separation and stacking matrices were added to the assigned wells according to the manufacturer’s protocol. The microplate was then placed in the AbbyTM instrument, and the fully automated electrophoresis and immunodetection were performed. The device’s camera detected chemiluminescence and produced digital images. Details of the antibodies used are provided in Supplementary Table S1, and uncropped virtual blots are presented in Supplementary Figure S1. Protein expression was normalized to β-actin as a loading control, and normalization calculations were performed using ProteinSimple Compass version 6.3.0.

### 2.10. Statistical Analysis

The data are presented as mean ± SEM from at least three independent experiments for all the assays. Data were analyzed using GraphPad Prism (version 9.4.1), and statistical significance was determined using one-way ANOVA followed by Dunnett’s multiple comparisons test (* *p* < 0.05, ** *p* < 0.01, *** *p* < 0.001, *** *p* < 0.0001, ns = not significant). Gene expression was quantified using CFX 3.1 Manager software (Bio-Rad), and protein expression was analyzed with Compass software 6.3.0 (ProteinSimple).

## 3. Results

### 3.1. Kaempferol Inhibits Cell Viability and Growth

**Figure 1:**
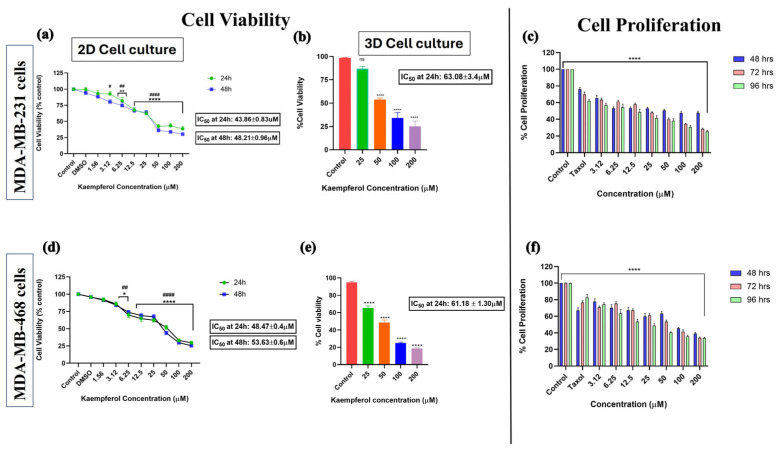
The effect of kaempferol on cell viability (2D and 3D cell culture) and on cell proliferation, and its comparison with the standard chemotherapeutic drug Taxol in MDA-MB-231 (**a**–**c**) and MDA-MB-468 (**d**–**f**) TNBC cells. The concentration of kaempferol varied from 1.56 to 200 µM, and the control cells were treated with DMSO (<0.1%). For the proliferation assay, the treatments used were: kaempferol (3.12–200 µM), Taxol (1 μM), and control (DMSO (<0.1%). The experiments were performed three times with n = 8 at 37 °C in 5% CO_2_. The data are presented as the mean ± SEM. Statistical significance between control vs. treatments was evaluated by a one-way ANOVA, followed by Dunnett’s multiple comparison tests. * *p* < 0.05, ** *p* < 0.01, **** *p* < 0.0001 (24 h) and # *p* < 0.05, ## *p* < 0.01, #### *p* < 0.0001, ns = *p* > 0.05 (48 h).

Cell viability was assessed using the Alamar Blue assay in 2D (24 and 48 h) and 3D (24 h) cell cultures. The cells were treated with different concentrations of kaempferol and the control (DMSO). A dose–response curve was observed with increasing concentrations of kaempferol. In the 2D culture of MDA-MB-231 cells, there was no significant difference in cell viability between the control and the lowest kaempferol concentration (1.56 μM). However, there was a significant difference in the percentage of viable cells between 3.12 μM and 200 μM of kaempferol compared to the control (Figure 1a). Similarly, in 3D cell culture for MDA-MB-231 cells, kaempferol exhibited a dose–response effect on cell viability. There was no significant difference in the % of cell viability between 25 μM and control; however, concentrations from 50 μM to 200 μM showed a dose-dependent inhibition of cell viability (Figure 1b). Likewise, in the 2D cell culture of MDA-MB-468 cells, there was no significant change in the % of cell viability between control and 1.56 μM of kaempferol, but there was a significant dose-dependent response from 3.12 μM to 200 μM of kaempferol as compared to control (Figure 1d). In the 3D cell culture of MDA-MB-468 cells, all the concentrations of kaempferol from 25 μM to 200 μM showed a significant dose-dependent decrease in the cell viability compared to the control (Figure 1e). The IC50 for MDA-MB-231 cells in 3D cell culture was approximately 1.5× higher (63.08 μM ± 3.4 μM) than in 2D cell culture (43.86 μM ± 0.83 μM at 24 h). Similarly, the IC50 calculated for MDA-MB-468 cells in 3D cell culture (61.18 μM ± 1.30 μM) was approximately 1.5× higher than in 2D cell culture (48.47 μM ± 0.4 μM), as expected. The IC50 values in 2D cell culture indicated that MDA-MB-231 cells are more sensitive to kaempferol than MDA-MB-468 cells at 24 h and 48 h. In 3D cell culture, cells were equally sensitive to kaempferol, as indicated by the IC50; however, the 468 cells were more sensitive at 25 µM.

The cell proliferation assay was conducted using Alamar Blue to assess kaempferol’s potential to inhibit the growth of both cell lines, compared with the standard chemotherapeutic drug Taxol at 1 μM. The antiproliferative activity of kaempferol was analyzed by measuring cell reduction of resazurin after incubation with kaempferol for 48, 72, and 96 h, and compared with Taxol. The analysis revealed a dose-dependent decrease in cell proliferation in both cell types, with kaempferol concentrations ranging from 3.12 μM to 200 μM. Kaempferol exhibited anti-proliferative effects similar to those of Taxol at 3.12 μM and higher for MDA-MB-231 cells (Figure 1c) and at 12.5 μM and higher for MDA-MB-468 cells (Figure 1f).

### 3.2. Kaempferol Induces Apoptosis

**Figure 2:**
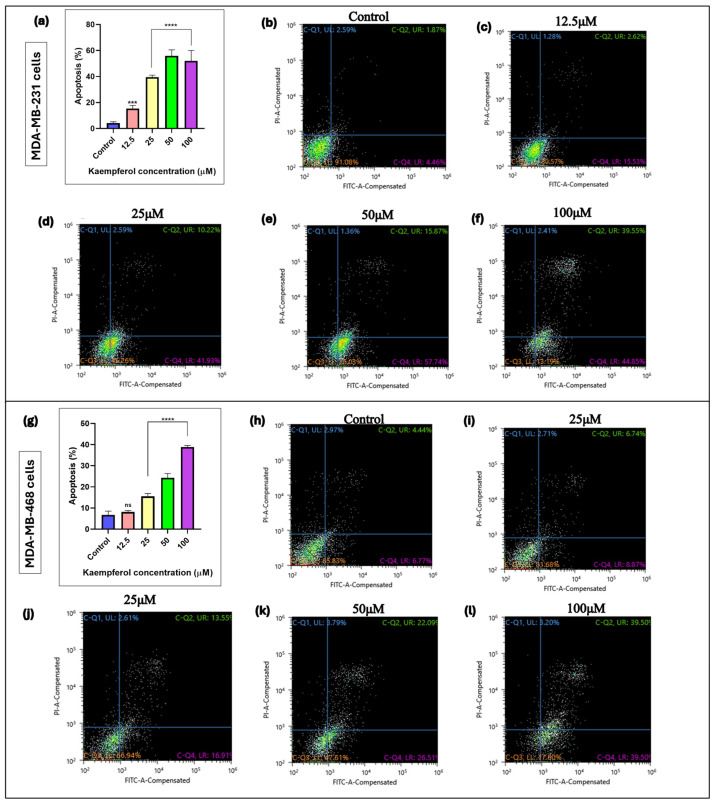
Effect of kaempferol to induce apoptosis in MDA-MB-231 cells (**a**–**f**) and MDA-MB-468 cells (**g**–**l**). The cells were treated with kaempferol concentrations ranging from 12.5 to 100 µM. At 24 h, the apoptotic effect of kaempferol was analyzed using a Sony SH800 Cell Sorter (San Jose, CA, USA) and the CELLQuest software 2.1.6. The experiment was performed 3 times, and the data are presented as the mean ± SEM. The statistically significant differences between control and treatments were evaluated with one-way ANOVA and Dunnett’s multiple comparison tests. *** *p* < 0.001, **** *p* < 0.0001, ns = *p* > 0.05.

The annexin V-FITC assay was used to assess the kaempferol-mediated induction of apoptosis in MDA-MB-231 and MDA-MB-468 cells. The cells were analyzed 48 h after treatment, and apoptosis was quantified using a flow cytometer. In MDA-MB-231 cells, kaempferol showed induction of apoptosis from 12.5 μM to 100 μM (from 41.93% to 58.74%) (Figure 2a–f). However, in MDA-MB-468 cells, the induction of apoptosis was observed at kaempferol concentrations between 25 μM and 100 μM (from 26.51% to 39.50%), respectively (Figure 2g–l). Overall, kaempferol showed an apoptotic effect in MDA-MB-231 cells at a concentration lower than in MDA-MB-468 cells.

### 3.3. Effect of Kaempferol on Dual Acridine Orange/Ethidium Bromide (AO/EtBr) Fluorescent Staining Assay

**Figure 3:**
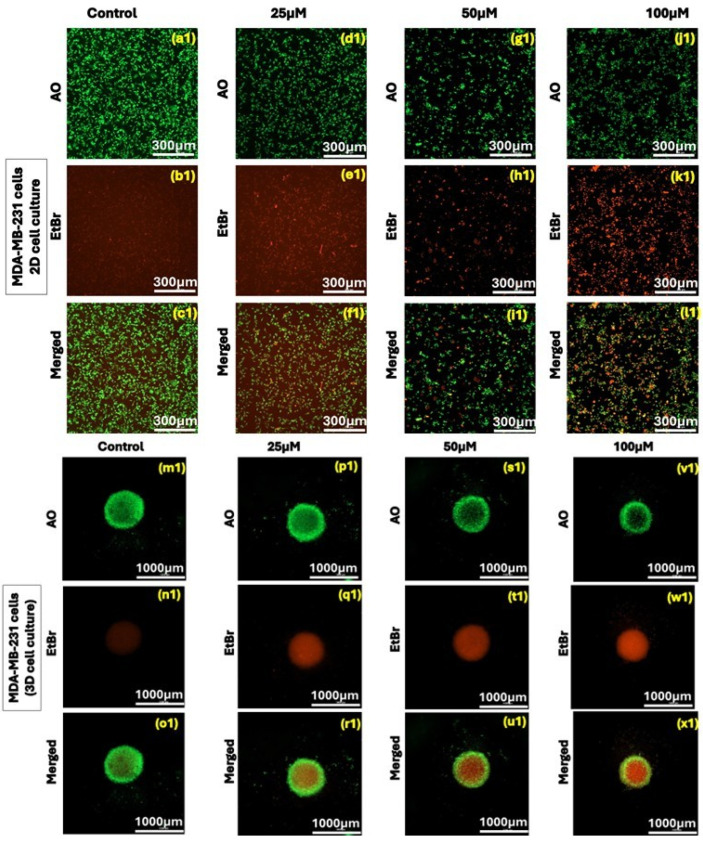

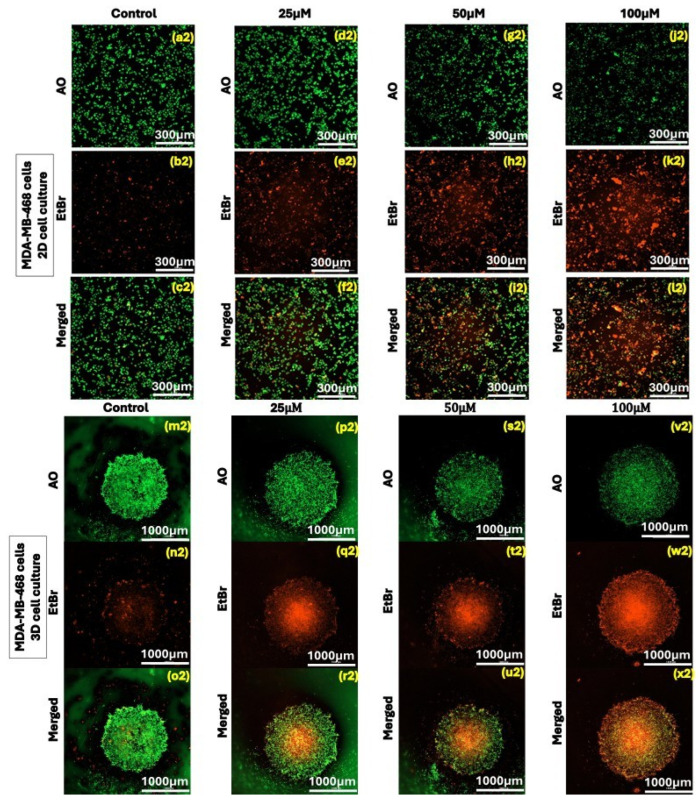
Effect of kaempferol on dual Acridine Orange/Ethidium bromide (AO/EtBr) fluorescent staining on MDA-MB-231 in 2D (**a1**–**l1**) and 3D (**m1**–**x1**) and on MDA-MB-468 cells in 2D (**a2**–**l2**) and 3D (**m2**–**x2**) cell culture. The cells were treated with a range of kaempferol concentrations, from 25 μM to 100 μM. Cell imaging was performed at 24 h and visualized using the Olympus Cell Sens Standard Cytation 5 cell Imaging reader (BioTek Instruments, Inc., Winooski, VT, USA) at 40× magnification.

The acridine orange/ethidium bromide (AO/EtBr) staining-based fluorescent assay was used to assess the effect of kaempferol concentrations (12.5 µM to 50 µM) on apoptosis induction in 2D and 3D cell cultures using MDA-MB-231 and MDA-MB-468 cells. The green fluorescence indicated healthy cells, and the red or orange colored fluorescence indicated apoptotic or early necrotic cells. The merged green and orange fluorescence showed increased apoptosis induction in both 2D and 3D cell cultures. In MDA-MB-231 cells, kaempferol treatment showed a significant decrease in the green, fluorescent cells from 25 μM to 100 µM (Figure 3(a1–x1)). Similarly, in MDA-MB-468 cells, kaempferol treatment induced apoptosis at concentrations above 25 µM, compared with control cells in both 2D and 3D cultures (Figure 3(a2–x2)). The results correlate with the ones obtained from the flow cytometry-based apoptosis assay.

### 3.4. Kaempferol Induces Cell Cycle Arrest

**Figure 4:**
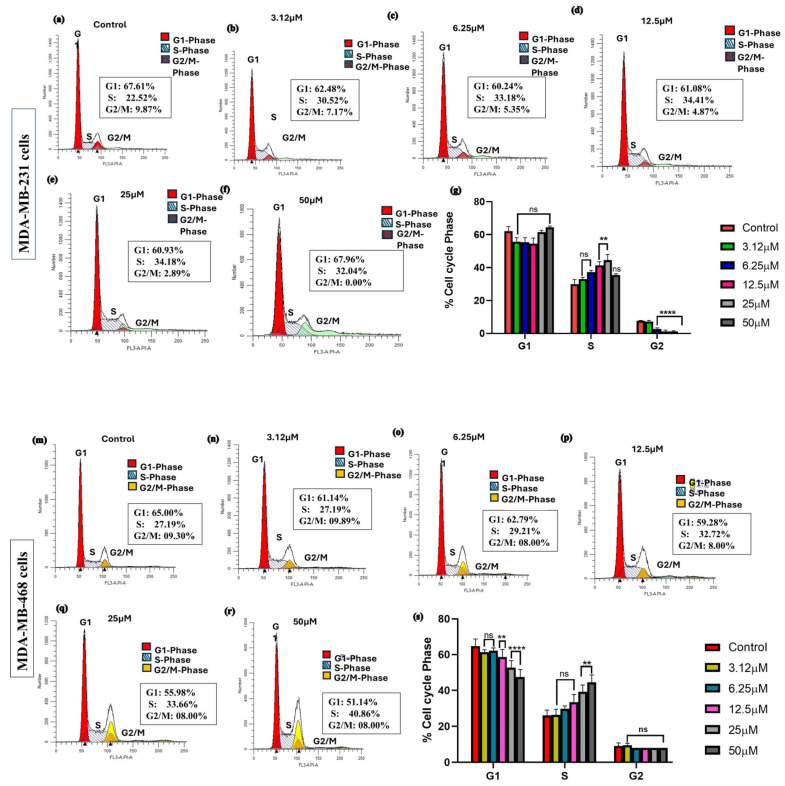
Effect of kaempferol on the induction of cell cycle arrest in MDA-MB-231 and MDA-MB-468 cells. The cells were treated with DMSO (control) and various concentrations of kaempferol, ranging from 3.12 to 50 µM, for 24 h. The ability of kaempferol to induce cell cycle arrest was analyzed using a Sony SH800 Cell Sorter (San Jose, CA, USA) and the CELLQuest software. The effect of kaempferol on the induction of cell cycle arrest is shown in MDA-MB-231 cells (**a**–**f**) and MDA-MB-468 cells (**g**–**l**). The data are presented as the mean ± SEM, and the statistical significance of the effect between control and treatments was evaluated by one-way ANOVA, followed by Dunnett’s multiple comparison tests.** *p* < 0.01, **** *p* < 0.0001, ns = *p* > 0.05.

A range of concentrations from 3.12 μM to 50 μM was used to investigate the effect of kaempferol on cell cycle arrest at 48 h in both MDA-MB-231 and MDA-MB-468 cells. The cell distribution among different cell cycle phases was analyzed using a Sony SH800 cell sorter and flow cytometer. In MDA-MB-231 cells, kaempferol induced a significant cell cycle arrest at the S phase at concentrations of 12.5 μM and 25 μM, compared to the control cells, with a significant decrease in the cell number in the G2 phase. At the lower concentrations of 3.12 and 6.25 μM, as well as the highest concentration of 50 μM, there was no statistically significant difference compared to the control (Figure 4a–f). In MDA-MB-468 cells, a significant increase in the number of cells in the S phase was observed with 25 μM and 50 μM of kaempferol compared to the control cells (Figure 4g–l). This change in the S phase was also accompanied by a significant concentration-dependent decrease in the percentage of cells in the G1 phase, from 12.5 μM to 50 μM, compared to the control cells. Overall, in both cell lines, kaempferol induced cell cycle arrest at the S phase of the cell cycle. MDA-MB-231 cells were more sensitive to kaempferol, causing arrest at 12.5 μM, compared to 25 μM in MDA-MB-468 cells.

### 3.5. Kaempferol Modulates the mRNA and Protein Expression of CDKs

**Figure 5:**
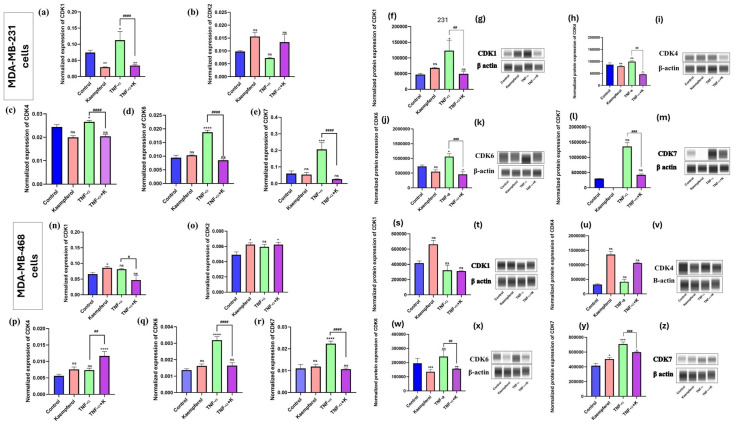
The effect of kaempferol on the mRNA expression levels of CDKs (CDK1, CDK2, CDK4, CDK6, and CDK7), and protein expression levels of CDK1, CDK4, CDK6, and CDK7 were evaluated in MDA-MB-231 (**a**–**m**) and MDA-MB-468 (**n**–**z**) cells using RT-PCR and Abby protein analysis, respectively. The MDA-MB-231 cells were treated with 25 μM of kaempferol, and the MDA-MB-468 cells with 50 μM of kaempferol, with or without TNF-α (50 ng/mL), and the control cells were treated with DMSO (<0.1%). The experiments were performed three times with n = 3 and incubated at 37 °C and 5% CO2. The data are presented as the mean ± SEM. The statistical significance between the control and treatments was evaluated using one-way ANOVA, followed by Dunnett’s multiple comparison tests. * *p* < 0.05, ** *p* < 0.01, *** *p* < 0.001, **** *p* < 0.0001 (control vs. TNF-α), and # *p* < 0.05, ## *p* < 0.01, ### *p* < 0.001, #### *p* < 0.0001 (TNF-α vs. co-treatment), ns = *p* > 0.05. The uncropped blots are shown in Supplementary Figure S1.

To further evaluate the effect of kaempferol on cell cycle in TNBC cells and determine the effect of kaempferol against mRNA expression of CDKs, the mRNA expression levels of CDK1, CDK2, CDK4, CDK6, and CDK7 were quantified by RT-PCR, and the protein expression of CDK1 and CDK7 was analyzed using Abby expression analysis. The effect of kaempferol on the mRNA expression of CDKs was studied using RT-PCR with individual primers for CDK1, CDK2, CDK4, CDK6, and CDK7. In MDA-MB-231 cells, co-treatment of kaempferol and TNF-α significantly downregulated the mRNA levels of CDK1 (4× fold), CDK7 (8.3× fold), CDK4 (1.2× fold), and CDK6 (1.9× fold) compared to TNF-α-only-treated cells (Figure 5a–e). The effect of TNF-α stimulation and kaempferol treatment was also analyzed for the mRNA expression levels of CDK2. However, there was no significant stimulation of CDK2 mRNA levels after TNF-α treatment. The co-treatment of TNF-α and kaempferol resulted in CDK2 levels higher than those of the control (Figure 5b). In MDA-MB-468 cells, the results showed that stimulation with TNF-α induced the expression of CDK6 and CDK7 (Figure 5q,r) significantly compared to the control. Meanwhile, in MDA-MB-468 cells, the co-treatment of TNF-α and kaempferol significantly downregulated the expression of CDK6 (1.9× fold) and CDK7 (2.1× fold) at mRNA levels (Figure 5n,r), as compared to the TNF-α-only-treated cells. Conversely, TNF-α stimulation did not induce the expression of CDK1, CDK2, and CDK4 (Figure 5n–p). However, with the co-treatment of TNF-α and kaempferol, only CDK1 showed levels lower than those of the control.

Based on the RT-PCR results, CDK1, CDK4, CDK6, and CDK7 were selected for further investigation at the protein level. In the MDA-MB-231 cells, the co-treatment of TNF-α and kaempferol decreased the CDK1 protein expression by 2.5× fold, CDK4 by 1.25× fold, CDK6 by 2×, and CDK7 by 3× fold, compared to cells treated with TNF-α only (Figure 5f–m). In the MDA-MB-468 cells, the co-treatment downregulated CDK6 protein levels by 1.25× fold (Figure 5w,x) and CDK7 by 1.2× fold (Figure 5y,z), compared to the cells treated with TNF-α only. However, with CDK1 and CDK4, the co-treatment showed no statistically significant difference compared to TNF-α only (Figure 5s–v). Overall, the protein level results confirm those at the gene level, demonstrating kaempferol’s ability to modulate different CDKs and its differential effects on both cell lines.

### 3.6. Effect of Kaempferol on mRNA Expression and Protein Release of PD-L1 and Its Inducers

**Figure 6:**
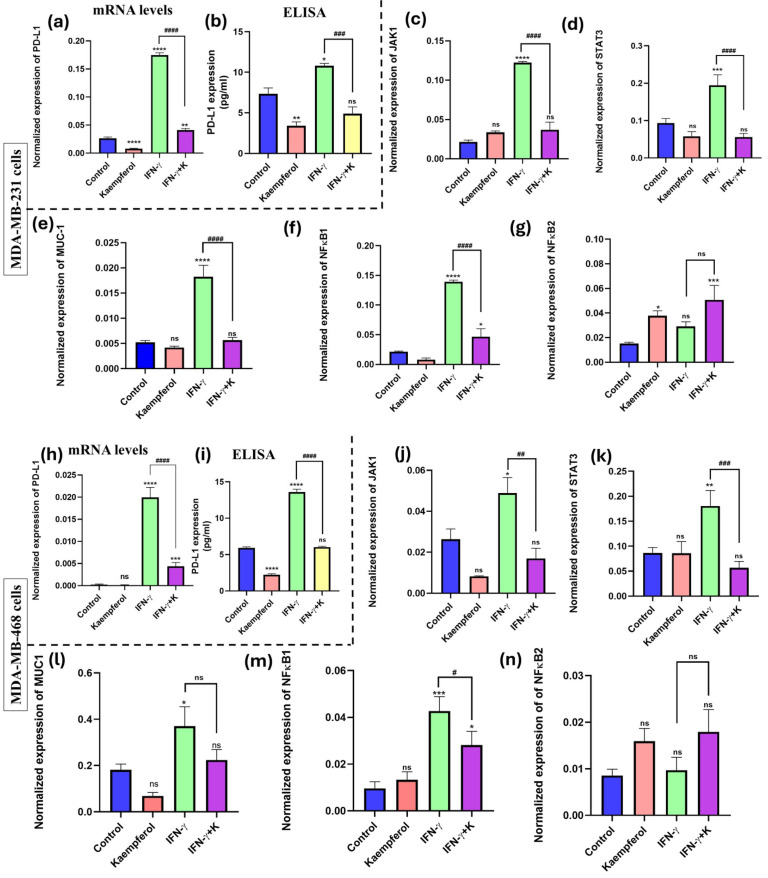
Effect of kaempferol on mRNA expression and protein release levels of PD-L1 and on gene expression of its inducers (JAK1, STAT3, MUC-1, NF-κB1, and NF-κB2) in TNBC cell lines. The cells were treated with 25 μM and 50 μM kaempferol in MDA-MB-231 and MDA-MB-468 cells, respectively, with or without IFN-γ (100 ng/mL). The control cells were treated with DMSO (<0.1%). The experiments were performed three times with n = 3 and incubated at 37 °C and 5% CO_2_. The data are presented as the mean ± SEM. The effect of kaempferol on the mRNA and protein release levels of PD-L1 is shown for MDA-MB-231 cells (**a**,**b**) and MDA-MB-468 cells (**h**,**i**). The effect of kaempferol on the mRNA levels of JAK1, STAT3, MUC-1, NFκB1, and NFκB2 is shown for MDA-MB-231 (**c**–**g**) and for MDA-MB-468 cells (**j**–**n**). The statistical significance between the control and treatments was evaluated using one-way ANOVA, followed by Dunnett’s multiple comparison tests. * *p* < 0.05, ** *p* < 0.01, *** *p* < 0.001, **** *p* < 0.0001 (control and TNF-α), and # *p* < 0.05, ## *p* < 0.01, ### *p* < 0.001, #### *p* < 0.0001 (TNF-α and co-treatment), ns = *p* > 0.05.

To investigate the effect of kaempferol on the mRNA expression and protein release of PD-L1, RT-PCR assay using a specific primer and an ELISA kit for PD-L1 were performed. The data showed that the stimulation with IFN-γ significantly induced the levels of PD-L1 mRNA expression (about 20-fold in both cell lines) (Figure 6a,h). It also showed a significant increase in protein release (about 12-fold) as compared to the control in both MDA-MB-231 and MDA-MB-468 cells (Figure 6b,i). The co-treatment of IFN-γ and kaempferol showed significant downregulation in the mRNA expression of PD-L1 (about 4-fold) in both cell lines. Similarly, the co-treatment showed significant downregulation of PD-L1 protein release in MDA-MB-231 (2-fold) (Figure 6a,b) and MDA-MB-468 (2.5-fold) cells (Figure 6h,i), compared to IFN-γ stimulation alone. Therefore, the data show that kaempferol significantly inhibited the expression of PD-L1 at both mRNA and protein levels in TNBC cells.

The effect of kaempferol on inducers of PD-L1 expression was analyzed using an RT-PCR assay with specific primers for JAK1, STAT3, MUC-1, NF-κB1, and NF-κB2. The treatment with IFN-γ significantly induced the expression of all genes, except for NF-κB2, in both MDA-MB-231 and MDA-MB-468 cells. The co-treatment of kaempferol and IFN-γ in MDA-MB-231 cells prompted a statistically significant decrease in the mRNA expression of JAK1, STAT3, MUC-1, and NF-κB1, as compared to the IFN-γ treatment alone (Figure 6c–g). In MDA-MB-468 cells, the co-treatment of IFN-γ and kaempferol resulted in a significant decrease in the mRNA expression of JAK1, STAT3, and NF-κB1; however, the change in MUC-1 levels was not significant (Figure 6j–n). Overall, the treatment of kaempferol revealed that the reduction in PD-L1 expression in both TNBC cell lines may be mediated via the JAK1/STAT3 signaling pathway and by the inhibition of MUC-1 levels and NF-κB1, which are involved in the transcription activation of PD-L1.

### 3.7. Kaempferol Reduced CCL2 and TGM2 mRNA Expression and Protein Release of CCL2

**Figure 7:**
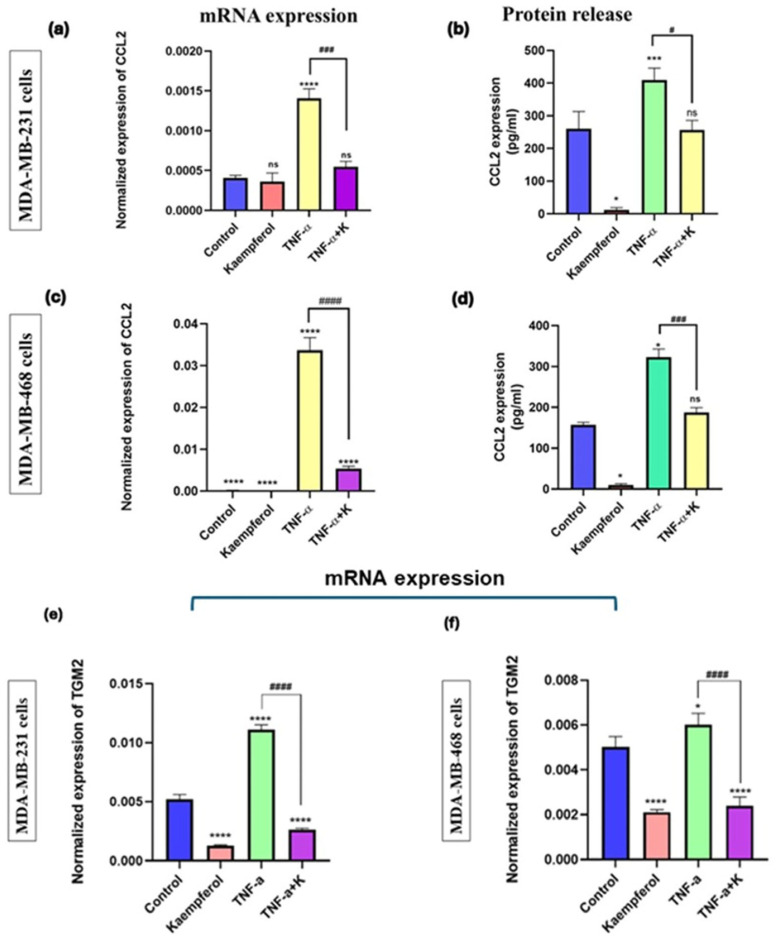
The effect of kaempferol on mRNA expression of CCL2 and TGM2 and protein release levels of CCL2 in MDA-MB-231 and MDA-MB-468 cells was assessed using RT-PCR analysis and ELISA, respectively. The cells were treated with kaempferol concentrations of 25 μM and 50 μM in MDA-MB-231 and MDA-MB-468 cells, respectively, with or without TNF-α (50 ng/mL). The control cells were treated with DMSO (<0.1%). The experiments were performed three times with n = 3 and incubated at 37 °C and 5% CO_2_. The data is presented as the mean ± SEM. The effect of kaempferol on mRNA expression and protein release levels of CCL2 is shown in MDA-MB-231 (**a**,**b**) and MDA-MB-468 cells (**c**,**d**). The effect of kaempferol on mRNA expression levels of TGM2 is shown in MDA-MB-231 and MDA-MB-468 cells in (**e**) and (**f**), respectively. The statistical significance between the control and treatments was evaluated using one-way ANOVA, followed by Dunnett’s multiple comparison tests. * *p* < 0.05, *** *p* < 0.001, **** *p* < 0.0001 (control vs. TNF-α), and # *p* < 0.05, ### *p* < 0.001, #### *p* < 0.0001 (TNF-α vs. co-treatment), ns = *p* > 0.05.

The effect of kaempferol on CCL2 and TGM2 mRNA expression, as well as on the CCL2 protein release level, was investigated using specific primers and a CCL2 ELISA kit. The treatment with TNF-α successfully stimulated CCL2 and TGM2 mRNA expression and CCL2 protein release levels in MDA-MB-231 and MDA-MB-468 cells, compared to the control (Figure 7a–f). The co-treatment of TNF-α and kaempferol significantly reduced CCL2 (2-fold) and TGM2 (3-fold) mRNA expression levels and protein release (5-fold) in MDA-MB-231 cells, as compared to the TNF-α treatment alone (Figure 7a,b,e). In MDA-MB-468 cells, the co-treatment significantly decreased the CCL2 (2.8-fold) and TGM2 mRNA expression (2.3-fold), and CCL2 protein release levels (5-fold) (Figure 7c,d,f). The data show that kaempferol is more effective in inhibiting CCL2 mRNA expression levels in MDA-MB-468 compared to MDA-MB-231 cells and has a similar downregulatory effect on CCL2 protein release levels in both TNBC cell lines.

## 4. Discussion

The role of TIME in TNBC development, progression, and treatment outcomes has garnered considerable attention in the last few decades [15]. Recent studies have shown the potential of natural compounds, specifically flavonoids, in modulating the TIME by modulating ROS-scavenging enzyme activities, inducing cell cycle arrest, promoting apoptosis, modulating autophagy, suppressing proliferation and invasiveness of cancer cells, and altering signaling pathways that are overactivated during the development and progression of cancer [16]. The flavonoid kaempferol has been primarily studied in MDA-MB-231 cells exhibiting diverse molecular anticancer mechanisms. Kaempferol has been demonstrated to target anti-oxidative stress and anti-inflammatory pathways, induce apoptosis, autophagy, and cell cycle arrest, and provide information on the effect of kaempferol on specific targets that could help to overcome the drug resistance observed with current immunotherapies. Also, not much is known about its anticancer effects on MDA-MB-468 cells, which can have a huge impact since the patient’s genetic tumor profile diversity has been proposed to contribute to TNBC disparities, and it can help evaluate how genetically different cells may respond to kaempferol treatment [17].

The data of this study showed that kaempferol exhibits cytotoxic and anti-proliferative effects in both MDA-MB-231 and MDA-MB-468 cells. This anti-proliferative effect observed in MDA-MB-231 cells is consistent with a study which also demonstrated a dose-dependent decrease in proliferation [18]. However, the IC50 values of both TNBC cells in 2D cell culture at 24 h and 48 h showed that MDA-MB-231 cells were more sensitive to kaempferol treatment than MDA-MB-468 cells. Conversely, in 3D cell culture, both cell lines exhibited equal sensitivity to the compound. Moreover, the cell proliferation assay showed a dose-dependent decrease in the cell growth of both cell lines at 48, 72, and 96 h. Additionally, it was observed that kaempferol produced a similar antiproliferative effect to the standard drug Taxol with lower concentrations in MDA-MB-231 compared to MDA-MB-468 cells. The cell antiproliferative effect of kaempferol demonstrates its potential to inhibit the aggressive tumorigenic nature of TNBC cells, thereby preventing them from proliferating, growing, and translating to disease aggressiveness [19,20,21].

Cell cycle regulators mediate cell proliferation and apoptosis processes. The misregulation of the cell cycle checkpoints leads to the induction of apoptotic cell death, featured in the form of DNA and nuclear fragmentation, mRNA decay, chromatin aggregation, and apoptotic bodies. The evasion of apoptosis due to genetic alterations leads to uncontrolled cell proliferation and an increase in tumor mass, invasion, and metastasis [22]. Multiple studies have demonstrated that kaempferol induces apoptosis, a major mechanism for its anti-cancer effect. In another study, authors reported that kaempferol induces caspase-9 and caspase-3 activation and increases γ-H2AX in MDA-MB-231 TNBC cells [18]. Similar findings showcasing induction of apoptosis and evaluation of the apoptotic markers with kaempferol treatment have been reported in other human cancer cells as well [23,24,25]. In this study, an apoptosis assay was performed in both TNBC cell lines. The results showed that kaempferol induced a significant increase in the percentage of apoptotic cells at concentrations of 12.5 μM and above in MDA-MB-231 cells. However, in the MDA-MB-468 cells, an increase in apoptotic cell number was observed at concentrations of 25 μM and above. Further, the induction of apoptosis in 2D and 3D cell cultures was confirmed using AO/EtBr staining assay. The results showed the induction of apoptotic cell death in both cell types, where kaempferol treatment increased the number of dead cells as the concentration of the compound was increased in both 2D and 3D cell cultures. Moreover, this study showed that kaempferol induced cell cycle arrest at the S phase of the cell cycle in both cell lines. The effect was observed between the concentrations of 12.5 μM and 25 μM in MDA-MB-231 cells, while in MDA-MB-468 cells, the arrest was observed at concentrations of 25 μM and 50 μM, indicating a different response from the cell lines studied. Notably, kaempferol has previously been reported to induce cell cycle arrest at G2/M in MDA-MB-231 cells and this difference likely reflects the concentration and time-dependent nature of kaempferol’s on the cell cycle checkpoints [18]. Higher kaempferol concentrations and longer exposures, as observed in Zhu et al. (2019), preferentially activate the G2/M checkpoint [18]. In contrast, the lower concentrations and 24 h treatment employed in the present study capture an earlier replication-associated block, leading to S-phase accumulation.

The progression of the cells through different phases in the cell cycle is controlled by cyclin-dependent kinases (CDKs) [26]. Most of these CDKs are upregulated in cancer tissues, leading to dysregulation of the cell cycle machinery [27]. The prominent CDK4/6 and their associated Cyclin D proteins are involved in the transition from G1 to S phase of the cell cycle. The FDA has approved three different CDK4/6 inhibitors to treat ER+/HER-2 breast cancer, but these treatments have inefficient results in TNBC [28]. Apart from CDK4/6, CDK2 is also involved in cell cycle progression through the G1, S, and G2 phases, and aberrant CDK2 activation is associated with CDK4/6 inhibitors in HER2-responsive breast cancers [29].

Additionally, the overexpression and dysregulation of CDK1 in human cancers support targeting CDK1 as a potential therapeutic [27,30]. In the present study, the effect of kaempferol on the overexpression of CDKs, including CDK1, CDK2, CDK4, CDK6, and CDK7, was evaluated. TNF-α-mediated activation of CDKs was used to recapitulate the elevated CDKs levels observed in TNBC cells. Kaempferol treatment significantly downregulated the mRNA levels of CDK1, CDK6, and CDK7 in the MDA-MB-231 cells, and only CDK6 and CDK7 were downregulated in MDA-MB-468 cells. At the protein level, CDK1 and CDK7 confirmed the transcriptional results in MDA-MB-231 cells; however, in MDA-MB-468 cells, only CDK7 showed decreased levels. According to the literature, CDK7 has also emerged as a promising target in different molecular types of breast cancer, including TNBC. Unlike other CDKs, CDK7 functions as a transcription regulator, initiating transcription by phosphorylating RNA polymerase II [26]. The CDK7 inhibition and its combinatorial therapy with estrogen therapy are considered an effective strategy in overcoming the treatment resistance [31]. Therefore, this study’s findings demonstrate kaempferol’s potential to inhibit CDK1 and CDK7, underscoring its importance as an important strategy in TNBC treatment. Notably, the differences observed between mRNA and protein expression, particularly for CDK1 and CDK7, in our study can be attributed to regulatory checkpoints downstream of transcription [32,33]. In studies by Vogel et al. and Schwanhäusser et al., the authors have demonstrated that CDK protein levels are heavily shaped by translation efficiency, phosphorylation-dependent stabilization/degradation, ubiquitin–proteasome turnover, and cyclin partner assembly [32,34]. For example, stabilizing CDK1–Cyclin B complexes, or TFIIH-associated CDK7 in its CAK module, can preserve protein pools even when transcript levels are suppressed [32]. Additionally, polyphenols, including kaempferol, can also influence the ubiquitin–proteasome system, altering the degradation of specific proteins [35,36,37]. For instance, kaempferol promoted Ubiquitin-Proteasome System (UPS)-mediated degradation of DNA methyltransferase 3B (DNMT3B), which can result in mRNA–protein mismatches [37,38]. These post-transcriptional and post-translational mechanisms have the potential to contribute to the mRNA–protein divergence seen in this study. Additionally, in this study, kaempferol downregulated the mRNA and/or protein levels of CDK1, CDK4, CDK6, and CDK7, indicating modulation of expression of these CDKs. To fully understand the effect of kaempferol on the inhibition of CDKs mentioned above, future studies incorporating biochemical kinase assays, target engagement analyses, and binding studies will demonstrate whether kaempferol also functions as a direct inhibitor of CDK catalytic activity.

Furthermore, the pan CDK inhibitors and CDK4/6 inhibitors have also been associated with significant systemic toxicity in various human cancers, especially in hormone-responsive, HER-2 negative, and metastatic breast cancer [39,40]. The poor selectivity and high toxicity towards normal cells have prevented various CDK inhibitors from progressing to clinical use [39]. Further research led to the development of novel, selective CDK inhibitors, including CDK4/6 inhibitors, which were reported to have little or no toxicity towards normal human cells. The newer CDK inhibitors, including CDK7 and CDK9 inhibitors, have also been reported to be less sensitive or to have no effect on MCF-10a, a normal breast epithelial cell line [41,42].

Interestingly, kaempferol has also been evaluated in normal human breast epithelial cells; in a study, the authors found that kaempferol prevented tumorigenesis in an in vitro model of 17β-estradiol-treated MCF-10A cells and in an in vivo model of N-methyl-N-nitrosourea (NMU)-induced breast cancer compared with the control [43]. Consistent with this, kaempferol, when delivered as a niosome-encapsulated formulation, exhibited potent cytotoxic and anti-metastatic effects in MCF-7 cells but no significant toxicity in normal MCF-10A breast epithelial cells at the IC_50_ doses for MCF-7 cells [44]. In addition, most of the toxicities observed with CDK inhibitors, particularly the early pan-CDK inhibitors, were seen predominantly in hormone-responsive breast cancer subtypes. This ultimately led to the development of newer CDK inhibitors, which are now FDA-approved for patients with ER-positive and HER2-negative breast cancer [39,45]. Also, their efficacy in TNBC remains uncertain due to the associated development of resistance. Consequently, combining CDK inhibitors with other targeted therapies is being actively explored as a strategy to improve treatment outcomes [39,45], hoping to overcome resistance [45,46]. Precisely, the ability to target multiple CDKs in TNBC offers a real opportunity to explore it as part of combination strategies, either with other targeted therapies or alongside synthetic CDK inhibitors.

Moreover, CDK inhibition in TNBC has also been reported to prime TNBC for better outcomes with anti-PD-L1-based therapies. The combinatorial approach of CDK inhibitors and PD-L1 inhibitors reveals that CDK inhibition at a suboptimal dose promotes immune cell infiltration into tumors and may complement anti-PD-L1-based immunotherapy [47]. PD-L1 abundance has been reported to be regulated by CyclinD/CDK4/CDK6, and inhibition of CDKs with specific inhibitors can increase PD-L1 levels [48,49]. In another study, the authors reported that inhibiting CDKs, including CDK2, CDK4, CDK6, and CDK7, enhances the efficacy of PD-L1 inhibitors. The evaluation of the combinatory effect of CDK4/6 inhibitor (Abemaciclib), CDK2 inhibitor (Fedraciclib), and CDK7 inhibitor (Samuracciclib) on the efficacy of anti-PD-L1 immunotherapy in their in vitro and in vivo xenograft models of MDA-MB-231 and 4T1 cells [46] showed that the combination of CDK4/6 inhibitor and CDK7 inhibitor with anti-PD-L1 immunotherapy respectively, significantly inhibited tumor growth in the xenograft models [46].

In the tumor microenvironment, PD-L1 binds to its receptor, PD-1, inhibiting T-cell activation and is considered a key contributor to immune resistance. The anti-PD-L1/PD-1 antibodies modulate the activity of these tyrosine kinases, thereby activating downstream signaling pathways involved in the proliferation and differentiation of cytotoxic CD8+ T cells [50]. Numerous studies have evaluated the clinical performance of anti-PD-1 and anti-PD-L1 inhibition, yielding excellent results; however, the complex nature of the heterogeneous disease presents challenges, including the development of resistance and adverse events [50]. The anti-PD-L1/PD-1 immunotherapies, such as pembrolizumab and atezolizumab, have shown significant anti-tumor responses, a favorable safety profile, and prolonged progression-free survival in PD-L1-positive metastatic TNBC [51,52]. However, PD-L1 inhibitors are still considered ineffective, and more effective therapeutic strategies are needed to address the development of resistance in TNBC [50]. The current study evaluated the ability of the flavonoid kaempferol to modulate PD-L1 levels and its potential to reverse the development of resistance. To replicate the pathological levels of PD-L1 in TNBC, MDA-MB-231 and MDA-MB-468 cell lines were treated with IFN-γ, which physiologically is secreted by CD8+ T cells to induce PD-L1 expression [53,54,55]. Upon CD8+ T cell activation, released IFN-γ binds its receptors and activates the JAK/STAT signaling pathway, leading to IRF-1 activation and induction of PD-L1 expression on tumor cells, as reported in various human cancer models [53,54,55]. The present investigation showed that when IFN-γ-activated TNBC cells were treated with kaempferol, it significantly reduced PD-L1 levels. Moreover, the effect of kaempferol on the IFN-γ-mediated JAK1/STAT3 signaling pathway was investigated to understand better how the compound affects downstream signaling associated with PD-L1 upregulation. The results showed that kaempferol treatment inhibited the mRNA levels of JAK1 and STAT3. Multiple studies have shown the crucial role of the JAK/STAT3 pathway in the efficiency of PD-L1 immune checkpoint inhibition in various human cancers [14,56,57]. To further advance the effect of kaempferol on the molecular mechanisms involved in inhibiting the PD-L1, the compound’s effect on the levels of Mucin 1 (MUC-1) was also investigated. The transmembrane terminal subunit of MUC-1 (MUC-1-C) functions as an oncoprotein, and its gene amplification is linked to 40% of breast cancers [58]. MUC-1 in breast cancer cells is hypo-glycosylated, apically localized, and aberrantly expressed on more than 90% of the breast cancer cell membranes. The MUC-1-C subunit of MUC-1 activates the β-catenin/MYC, NF-κB pathway, and PI3/AKT signaling pathways. MUC-1 binds to p65 NF-κB subunit and forms MUC-1/p65 transcriptionally activated complex. This activation causes the NF-κB to enhance the MUC-1 expression further, thereby forming a MUC-1-NF-κB self-sustaining loop [6,58]. The present study showed that kaempferol treatment downregulates the mRNA expression of MUC-1 and NF-κB1 (p50) in both MDA-MB-231 and MDA-MB-468 cells, indicating that the inhibition of PD-L1 levels in TNBC cells may be via the inhibition of MUC-1 and subsequent reduction in the expression of NF-κB1 (p50). Therefore, kaempferol’s effect on the JAK/STAT pathway, as well as its inhibitory effects on MUC-1 and NF-κB1 expressions, indicates the potential of this compound to reverse the reduced tumor infiltration of T cells in the TIME.

NF-κB activation is also involved in the development of inflammation through the transcription of various chemokines such as CXCL2, CCL2, and IL-1β, which are involved in the recruitment of myeloid cells to the TIME [59,60]. CCL2, specifically, is a strong initiator of inflammation and is a chemokine attractant involved in the recruitment of macrophages within the TIME [61]. The neutralization of CCL2 with antibodies in the xenograft model of human breast cancer 4T1 cells and MDA-MB-231 cells showed a decrease in growth tumors and metastasis [4,10]. Similarly, in the xenograft mice models of breast cancer, the silencing of the CCL2 gene resulted in reduced tumor growth and metastasis [4]. CCL2 also blocks CD8+ T cell infiltration into tumor cells by binding to its CCR2 receptor on T cells [8]. Oncogenic pathways, including NF-κB and PI3K/AKT activation, that induce PD-L1 expression and PD-L1 immune checkpoint inhibitor resistance have been reported to induce CCL2 expression [8]. Another study also suggested that transglutaminase 2 (TG2), a post-translational modification enzyme, induced the expression of both PD-L1 and CCL2 by activating NF-κB and PI3/AKT pathways. They demonstrated that TG2 also induced PD-L1 immune checkpoint inhibitor resistance when evaluated in the presence of a PD-L1 inhibitor, indicating that CCL2 is another prominent regulator of resistance development, in addition to PD-L1 [8]. In the present study, kaempferol treatment in TNF-α-stimulated cells significantly decreased CCL2 mRNA levels in MDA-MB-231 and MDA-MB-468 cells, demonstrating that kaempferol inhibition of CCL2 and PD-L1 levels may enhance the efficacy of current PD-L1 inhibitors and help prevent resistance development.

**Figure 8:**
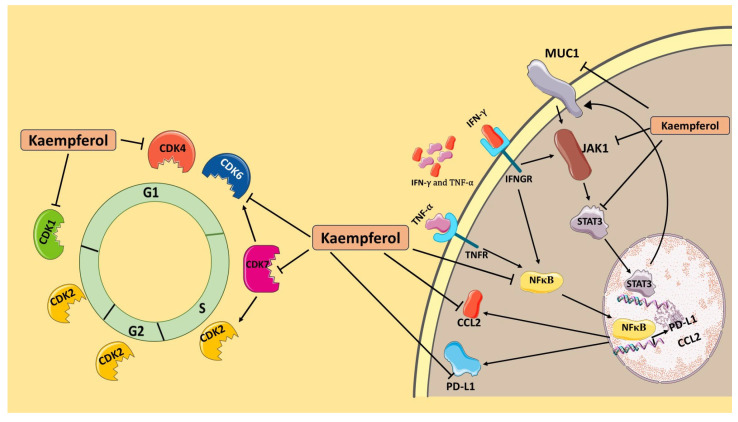
Kaempferol (K) inhibits multiple cell cycle CDKs (CDK1, CDK4/6, CDK7) and key PD-L1-regulatory nodes (JAK1, STAT3, MUC-1, NF-κB1, CCL2, TGM2) in TNBC cells, thereby suppressing proliferation and pathways associated with immune evasion and treatment resistance. The black arrow indicates induction of expression.

TGM2, a multifunctional GTPase overexpressed in various human cancers, is an oncogene that has also been reported to contribute to metastasis and drug resistance. TGM2 was reported to be overexpressed in PD-L1 and TNBC cells [62]. A 2020 study demonstrated that NFκB and PI3K/AKT activation induced by TGM2 are responsible for increased PD-L1 levels [8]. Elevated levels of TGM2, PD-L1, and CCL2 have also been reported in the TNBC patients. Due to increased expression of TGM2 and CCL2, along with PD-L1, in TNBC cells, immunotherapy targeting PD-1/PD-L1 immune checkpoints can develop resistance through evasion of the T cell immune response mediated by TGM2-induced CCL2 [62]. TGM2 has emerged as a novel diagnostic marker and can be used as a target to overcome the PD-1/PD-L1 inhibitor resistance [8]. Emerging evidence also suggests an important role for the aryl hydrocarbon receptor (AhR), a ligand-activated transcription factor involved in inflammatory signaling, immune evasion, and cell-cycle dysregulation [63]. Kaempferol has also been shown to function as an AhR ligand and to modulate AhR-dependent transcription of inflammatory signaling, immune evasion, and cell-cycle dysregulation, making it a relevant upstream regulator of several pathways assessed in this study [64]. Notably, AhR activation has been reported to regulate CDKs, enhance CCL2-mediated signaling, and regulate PD-L1 transcription [65]. Evaluating the contribution of AhR signaling to kaempferol-mediated modulation of these pathways is an important direction that to be assessed in future studies. In this study, kaempferol significantly downregulated the levels of TGM2 in both MDA-MB-231 and MDA-MB-468 cells, demonstrating that the simultaneous inhibition of PD-L1, CCL2, and TGM2 with kaempferol may be a novel strategy in adjuvant therapy to reverse treatment resistance in TNBC (Figure 8). Despite the promising multi-target anticancer effects of kaempferol observed in our study and consistent with prior literature, including our recent comprehensive review, kaempferol exhibits low aqueous solubility, extensive first-pass metabolism, and limited oral bioavailability, contributing to delays in its therapeutic development.

## 5. Conclusions

This study presents the molecular mechanisms by which the flavonoid kaempferol modulates the TIME by targeting multiple molecules and different signaling pathways. The data show that kaempferol presents cytotoxic and antiproliferative effects, induces apoptosis, and promotes cell cycle arrest at the S phase. Kaempferol also downregulated the expression of CDK1, CDK4, CDK6, and CDK7 in MDA-MB-231 cells and CDK6 and CDK7 in MDA-MB-468 cells at both the gene and protein levels, indicating that the compound could help improve the poor outcomes observed in TNBC associated with the overexpression of CDKs. Moreover, the data demonstrate that kaempferol shows significant inhibition of the mRNA and protein levels of PD-L1 in both TNBC cell lines. The underlying pathway may involve targeting the JAK1/STAT3 pathway and downregulating MUC-1 and NF-κB1 (p50). While inhibiting NF-κB1 levels, kaempferol also inhibited the overexpression of CCL2 and TGM2, which are responsible for the development of resistance to targeted therapies, such as anti-PD-L1 immunotherapy, in TNBC. This presents convincing evidence for further evaluation of kaempferol as an adjuvant therapy to overcome the development of treatment resistance in TNBC. Since PD-L1 levels have previously been shown to be regulated by CDKs, this study demonstrates that kaempferol inhibits PD-L1 expression. Therefore, the current study is the first to report the dual inhibitory effect of a natural flavonoid on CDKs and PD-L1 levels in TNBC cells. In conclusion, this study describes kaempferol’s anticancer effects through TIME modulation via various molecular mechanisms and targets, which may be essential in developing more effective combined therapies against TNBC.

## Data Availability

The original contributions presented in this study are included in the article/supplementary material. Further inquiries can be directed to the corresponding authors.

## Supplementary Materials

The following supporting information can be downloaded at: https://www.mdpi.com/article/10.3390/cancers17243911/s1, Table S1: Description of primers and antibodies used in this research; Figure S1: Uncropped virtual blots from Abby protein analysis.
