# Supplementary material for: The Anticancer Effect of Kaempferol Through Downregulation of CDKs and PD-L1 in Triple-Negative Breast Cancer Cells
Source: Cancers (Basel). 2025 Dec 6;17(24):3911. doi: 10.3390/cancers17243911 (PMC12730358; doi:10.3390/cancers17243911)

Supplementary Table S1: Description of primers and antibodies used in this research.

qPCR Primers:

**Target**	**Primer description**	**Brand**	**Unique Assay ID**
GAPDH	PrimePCR™ SYBR® Green Assay: GAPDH, Human	Bio-rad	qHsaCED0038674
JAK1	PrimePCR™ SYBR® Green Assay: JAK1, Human	Bio-rad	qHsaCID0008780
STAT3	PrimePCR™ SYBR® Green Assay: STAT3, Human	Bio-rad	qHsaCID0010912
PD-L1	PrimePCR™ SYBR® Green Assay: CD274, Human	Bio-rad	qHsaCID0036468
TGM2	PrimePCR™ SYBR® Green Assay: TGM2	Bio-rad	qHsaCID0007428
MUC1	PrimePCR™ SYBR® Green Assay: MUC1, Human	Bio-rad	qHsaCED0019841
NFκB1	PrimePCR™ SYBR® Green Assay: NFKB1	Bio-rad	qHsaCED0002379
NFκB2	PrimePCR™ SYBR® Green Assay: NFKB2	Bio-rad	qHsaCED0042395
CDK7	PrimePCR™ SYBR® Green Assay: CDK7	Bio-rad	qHsaCED0044530
CDK1	PrimePCR™ SYBR® Green Assay: CDK1, Human	Bio-rad	qHsaCID0036777
CDK2	PrimePCR™ SYBR® Green Assay: CDK2	Bio-rad	qHsaCED0003497
CDK4	PrimePCR™ SYBR® Green Assay: CDK4	Bio-rad	qHsaCED0003626

Abby Protein Analysis Antibodies:

Target	Antibody	Specificity / Sensitivity	Species Reactivity	Brand	Cat. No.	Dilution
CDK4	CDK4 (D9G3E)	Rabbit	Human	Cell Signaling Technology, Inc.	12790	1:125
CDK6	Anti-Cdk6 antibody [EPR4515]	Rabbit	Human	Abcam Limited	ab124821	1:125
CDK1	Anti-Cdk1 antibody [BLR085G]	Rabbit	Human	Abcam Limited	ab265590	1:125
CDK7	Anti-Cdk7 antibody [EPR23695-115]	Rabbit	Human	Abcam Limited	ab256787	1:125
B-actin	β-Actin Antibody	Rabbit	Human	Cell Signaling Technology, Inc.	4967	1:600

Supplementary Figure S1: Uncropped virtual blots from Abby protein analysis.

Unccropped Blots

MDA-MB-231 cells:

MDA-MB-231 CDK1:

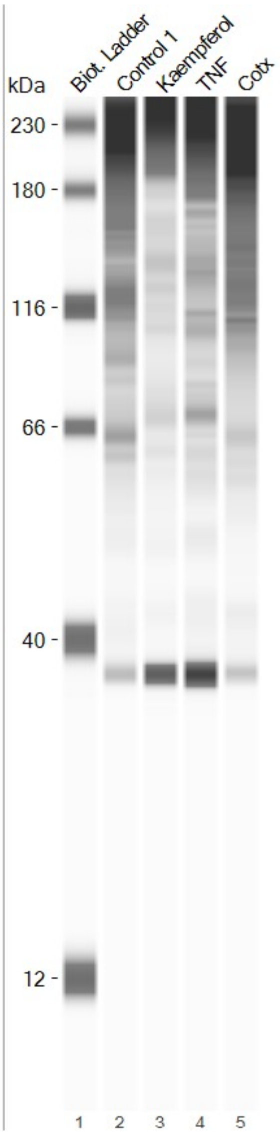

MDA-MB-231 CDK4:

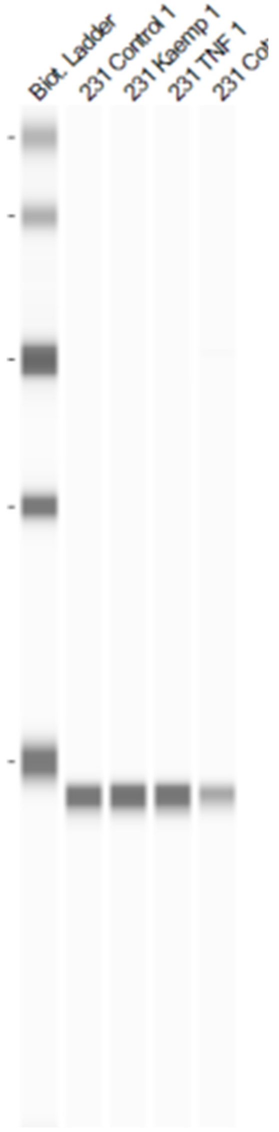

MDA-MB-231 CDK6:

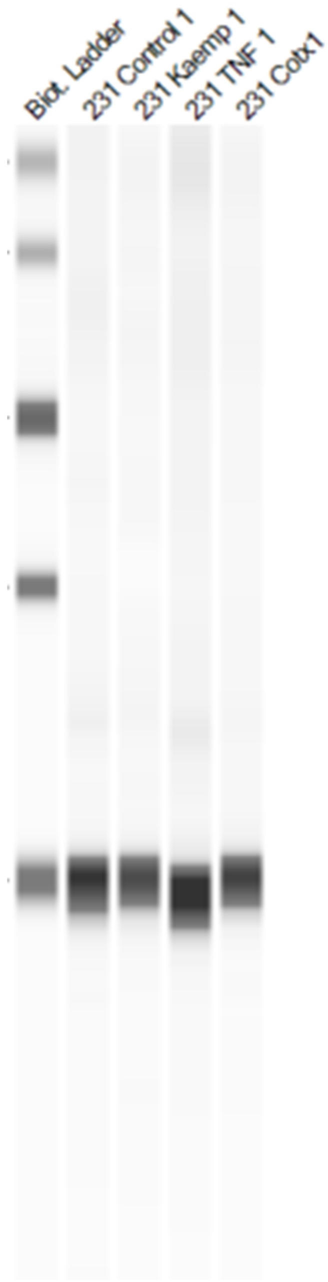

MDA-MB-231 CDK7:

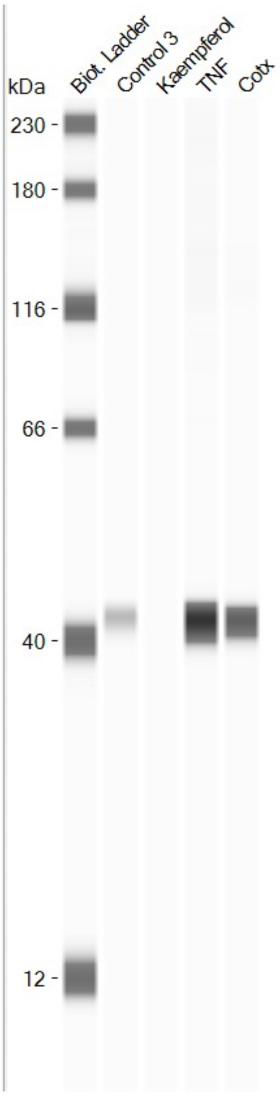
kDa
Biot. Ladder
Control 3
Kaempferol
TNF
Cotx
230
180
116
66
40
12

MDA-MB-468 cells:

MDA-MB-468 CDK1:

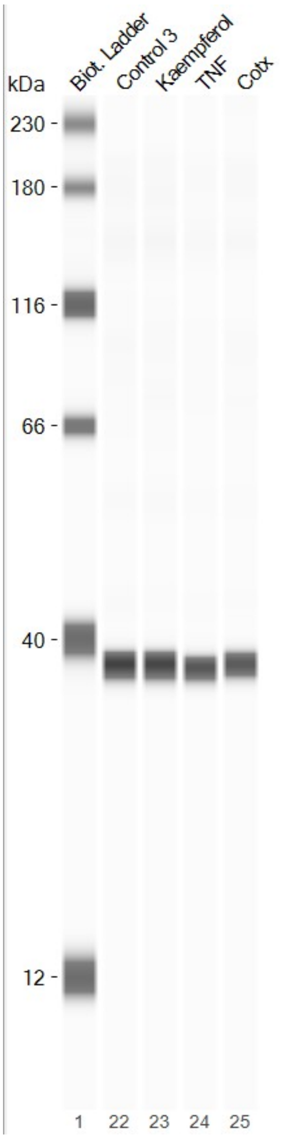

MDA-MB-468 CDK4:

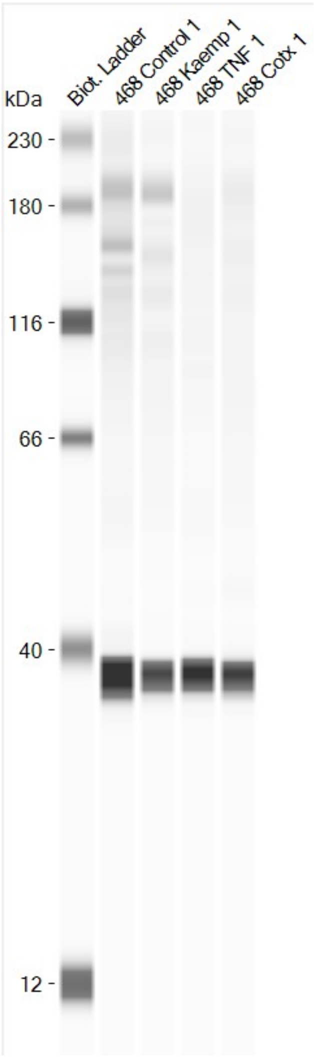

MDA-MB-468 CDK6:

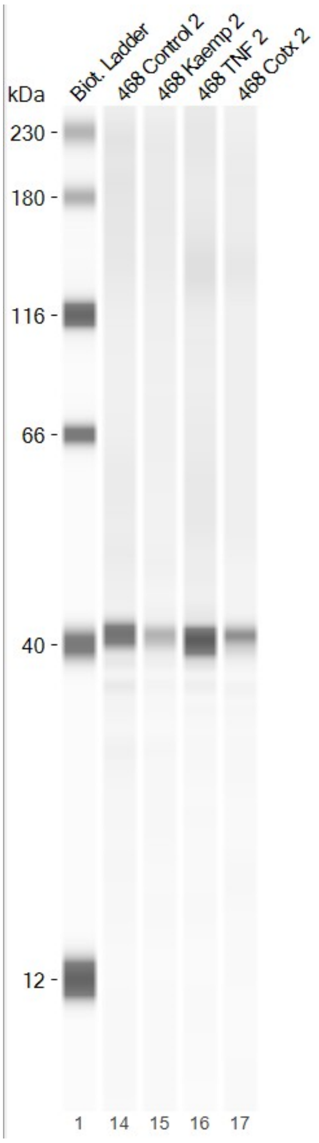

MDA-MB-468 CDK7:

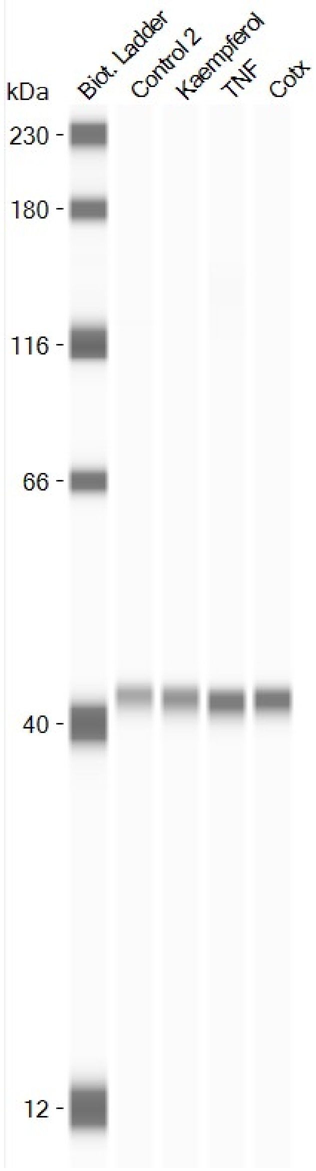

Supplement: Supplementary file 1 [file cancers-17-03911-s001.zip › cancers-4011148-supplementary.pdf]
